# Neuropilin2 regulates the guidance of post-crossing spinal commissural axons in a subtype-specific manner

**DOI:** 10.1186/1749-8104-8-15

**Published:** 2013-07-31

**Authors:** Tracy S Tran, Edward Carlin, Ruihe Lin, Edward Martinez, Jane E Johnson, Zaven Kaprielian

**Affiliations:** 1 Department of Biological Sciences, Rutgers University, Boyden 206, 195 University Ave., Newark, NJ 07102, USA; 2 Dominick P. Purpura Department of Neuroscience, Albert Einstein College of Medicine, Kennedy Center Room 624, 1410 Pelham Parkway South, Bronx, NY 10461, USA; 3 Department of Pathology, Albert Einstein College of Medicine, Kennedy Center Room 624, 1410 Pelham Parkway South, Bronx, NY 10461, USA; 4 Department of Neuroscience, University of Texas Southwestern Medical Center, Dallas, Texas, USA

**Keywords:** Semaphorins, Atoh1, Neurog2, Development, Spinal cord

## Abstract

**Background:**

Spinal commissural axons represent a model system for deciphering the molecular logic that regulates the guidance of midline-crossing axons in the developing central nervous system (CNS). Whether the same or specific sets of guidance signals control the navigation of molecularly distinct subtypes of these axons remains an open and largely unexplored question. Although it is well established that post-crossing commissural axons alter their responsiveness to midline-associated guidance cues, our understanding of the repulsive mechanisms that drive the post-crossing segments of these axons away from the midline and whether the underlying guidance systems operate in a commissural axon subtype-specific manner, remains fragmentary at best.

**Results:**

Here, we utilize axonally targeted transgenic reporter mice to visualize genetically distinct dorsal interneuron (dI)1 and dI4 commissural axons and show that the repulsive class 3 semaphorin (Sema3) guidance receptor Neuropilin 2 (Npn2), is selectively expressed on the dI1 population and is required for the guidance of post-crossing dI1, but not dI4, axons. Consistent with these observations, the midline-associated Npn2 ligands, Sema3F and Sema3B, promote the collapse of dI1, but not dI4, axon-associated growth cones *in vitro*. We also identify, for the first time, a discrete GABAergic population of ventral commissural neurons/axons in the embryonic mouse spinal cord that expresses Npn2, and show that Npn2 is required for the proper guidance of their post-crossing axons.

**Conclusions:**

Together, our findings indicate that Npn2 is selectively expressed in distinct populations of commissural neurons in both the dorsal and ventral spinal cord, and suggest that Sema3-Npn2 signaling regulates the guidance of post-crossing commissural axons in a population-specific manner.

## Background

Distinct populations of commissural neurons are widely distributed along the dorsoventral (D-V) and mediolateral (M-L) axes of the developing vertebrate spinal cord, and can be distinguished by their morphology, cell body position, gene expression patterns and axonal trajectories [1-6]. Although all commissural neurons extend axons across the floor plate (FP), an intermediate target at the ventral midline of the spinal cord, whether the same guidance signals control the pathfinding of each subtype towards (pre-crossing) and away (post-crossing) from the floor plate remains a largely unexplored issue.

The basic-helix-loop-helix (bHLH) transcription factors *Atoh1*, *Neurog1* and *Neurog2* define specific neuronal progenitor populations that give rise to genetically distinct dI1, dI2 and dI4 dorsal commissural neurons in the embryonic mouse spinal cord [7-9]. Enhancer elements derived from these bHLH factors direct reporter expression to distinct populations of commissural axons as they project toward, across and beyond the FP [10-16]. An antibody specific for GAD65, a rate-limiting enzyme required for GABA synthesis, labels ventral commissural neurons in the embryonic rat spinal cord [17,18]. Together, these markers provide tools for investigating molecular mechanisms that control the guidance of dorsal and ventral commissural axon subtypes.

Post-crossing commissural axons must lose and gain responsiveness to midline attractants and repellents, respectively, in order to successfully project away from the FP [19]. Repulsive signaling resulting from interactions between Robo receptors on post-crossing commissural axons and their Slit ligands on FP cells prevents multiple populations of commissural axons from re-crossing, and lingering at, the FP [16,19-22]. Inhibitory interactions between the Npn2 receptor and the ventral midline-associated Sema3s, Sema3B and Sema3F [23-25], facilitate the switch in responsiveness exhibited by post-crossing commissural axons [26-29]. However, it remains to be determined whether Sema3-Npn2 interactions regulate the pathfinding of all or only specific subsets of commissural axons.

In this study, we utilize transgenic reporter mouse lines that selectively label dI1 or dI4 dorsal commissural axons and anti-mouse GAD65 as a marker for ventral commissural axons to assess the role(s) of Sema3-Npn2 signaling in the pathfinding of these distinct axon populations. Whereas the *Atoh1-tauGFP* reporter has previously been shown to specifically label dI1 neurons [11,15], here, we demonstrate that a novel transgenic mouse line, *Neurog2-tauGFP*, targets GFP to a subset of the *Neurog2* expressing progenitors in the dorsal neural tube that give rise to dI4 commissural neurons. We find that dI1, but not dI4, commissural axons express Npn2, and require this receptor for navigating on the contralateral side of the ventral midline and for Sema3-mediated collapse of their growth cones *in vitro*. We also show, for the first time, that a GABAergic population of ventral commissural axons is present in the embryonic mouse spinal cord and that Npn2 regulates the guidance of their contralateral projections. Together, these findings indicate that Npn2 regulates the pathfinding of contralateral commissural projections in a subtype-specific manner.

## Results

### *Atoh1* and *Neurog2* enhancers drive GFP expression in spinal dI1 and dI4 commissural neurons, respectively, and dI1, but not dI4, neurons express Npn2

**Figure 1:**
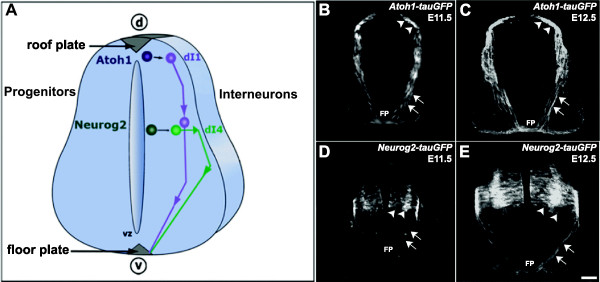
***Atoh1-tauGFP *and *Neurog2-tauGFP *transgenic mice direct GFP expression to distinct populations of spinal commissural neurons. (A)** Schematic of the embryonic mouse spinal cord in transverse view. In the developing mouse spinal cord, *Atoh1* or *Neurog2* progenitors give rise to dI1 and dI4 dorsal interneurons, respectively, that both project axons towards the floor plate (FP) and across the ventral commissure (VC). **(B, C)** In transverse cryosections derived from E11.5 and E12.5 *Atoh1-tauGFP* mouse embryos, tauGFP expression is present on a subset of dorsal progenitors, as well as on the dI1 interneurons that they differentiate into and their axons. **(D, E)** Cryosections from E11.5 and E12.5 *Neurog2-tauGFP* mice have tau-GFP localized to a distinct, more ventrally located subset of progenitors (arrowheads), as well as the dI4 interneurons that they differentiate into and their axons. Note that the pre-crossing segments of the labeled dI1 axons in **B** and **C**, project along a more medial route to the FP relative to their dI4 counterparts, in **D** and **E**, which project to the FP along a lateral trajectory. In both *Atoh1-tauGFP* and *Neurog2-tauGFP* mice, GFP expression is associated with pre- (arrows mark these axonal segments as they project toward the ventral midline) and post-crossing (asterisks mark these axonal segments as they project in the longitudinal plane within the marginal zone) segments of commissural axons. Arrowheads indicate positions of dI1 and dI4 cell bodies. Scale bar in **E**, 100 μm for **B**-**E**.

Dorsal interneurons in the developing mouse spinal cord arise from discrete populations of progenitors, which express particular basic helix-loop-helix (bHLH) transcription factors [5,30]. For example, *Atoh1-* and *Neurog1-*expressing progenitors differentiate into dI1 and dI2 neurons, respectively, in the developing spinal cord [7,8,11]. In addition, *Neurog2*-expressing progenitors give rise to dorsal spinal cord neurons in the dI2 and dI4 domains [5]. It has previously been shown that enhancer elements present within *Atoh1* and *Neurog2* loci are capable of driving reporter gene expression in either dI1 or dI4 neurons within the spinal cords of transgenic mice [10-15]. Furthermore, it has been demonstrated that the *Atoh1* enhancer element used in this study and a *Neurog1* enhancer element can direct tau-GFP to pre- and post-crossing dI1 and dI2 commissural axons, respectively, in chick embryos [16,31]. To elucidate molecular mechanisms that control the guidance of dI1 and dI4 commissural axons in mammals, we first characterized their trajectories in the spinal cords of *Atoh1-tauGFP* and *Neurog2-tauGFP* transgenic mouse lines (Figure 1A). In transverse spinal cord sections derived from *Atoh1-tauGFP* embryos at E11.5 and E12.5, GFP expression is present on dI1 neurons that occupy a narrow region extending from a location adjacent to the dorsal midline to a point midway along the dorsoventral axis of the spinal cord, as well as their axons, which project ventrally to, across, and on the contralateral side of the FP (Figure 1B,C). For this study, we generated *Neurog2-tauGFP* transgenic reporter mice in which the *Neurog2* gene is modified such that GFP expression is selectively directed to dI4 neurons (see Methods). In sections derived from *Neurog2-tauGFP* embryos at E11.5 and E12.5, GFP is expressed in dI4 neurons located within a broad domain of the dorsal spinal cord, and their ventrally projecting axons, which extend to, across, and on the contralateral side of the FP (Figure 1 D, E). Notably, GFP-labeled dI4 axons travel along a more lateral route to the FP than their dI1 counterparts (compare Figure 1 panels C,E with B,D), consistent with previously described dI4 axonal projections [13].

**Figure 2:**
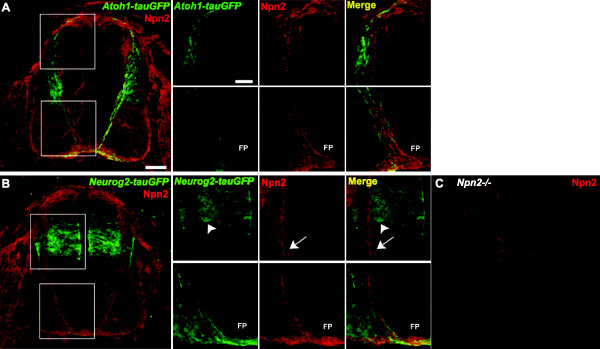
**Neuropilin 2 (Npn2) is selectively expressed on the cell bodies and axons of dI1 commissural neurons. (A)** A transverse spinal cord section derived from an E11.5 *Atoh1-tauGFP* mouse and labeled with anti-Npn2. The boxed regions were viewed at high magnification via confocal microscopy and these images are shown immediately to the right of panel **A**. There is significant overlap between tauGFP (green) and Npn2 (red) expression on dI1 cell bodies (upper panels) and all along their axons as they project to, across and on the contralateral side of the ventral midline (lower panels). **(B)** A transverse E11.5 mouse section shows that the *Neurog2-tauGFP* transgene is labeled with anti-Npn2. The boxed regions were examined at high magnification via confocal microscopy and these images are shown immediately to the right of panel **B**. The cell bodies of *Neurog2-tauGFP* expressing dI4 cells are distributed in the dorsal part of the spinal cord, but are located more ventrally (top box) than their dI1 counterparts. In contrast to the significant overlap observed for Atoh1-tauGFP and Npn2 expression, most Neurog2-derived dI4 interneurons and their pre-, midline- and post-crossing axons do not appear to express Npn2. **(C)**. A transverse spinal cord section taken from an E11.5 Npn2 null embryo was labeled with anti-Npn2. Scale bar in **A**, 100 μm for **A**-**C**; scale bar in **A** top-panel labeled with GFP, 25 μm for all boxed area panels for **A**-**B**.

To determine whether dI1 and dI4 neurons express Npn2, we labeled transverse cryosections derived from E11.5 *Atoh1-tauGFP* and *Neurog2-tauGFP* reporter mice with an anti-rat Npn2 polyclonal antibody. The specificity of this reagent for Npn2 was confirmed by showing that anti-Npn2 does not label spinal cord sections derived from *Npn2* null mouse embryos (Figure 2C). Whereas most or all GFP-labeled dI1 neurons and the pre-, midline- and post-crossing segments of their axons express Npn2 (Figure 2A), there is no significant overlap between Npn2 expression and GFP-labeled dI4 cell bodies/axons (Figure 2B).

### Pre-crossing and post-crossing segments of dI1 and dI4 commissural axons can be separately visualized by confocal microscopy

**Figure 3:**
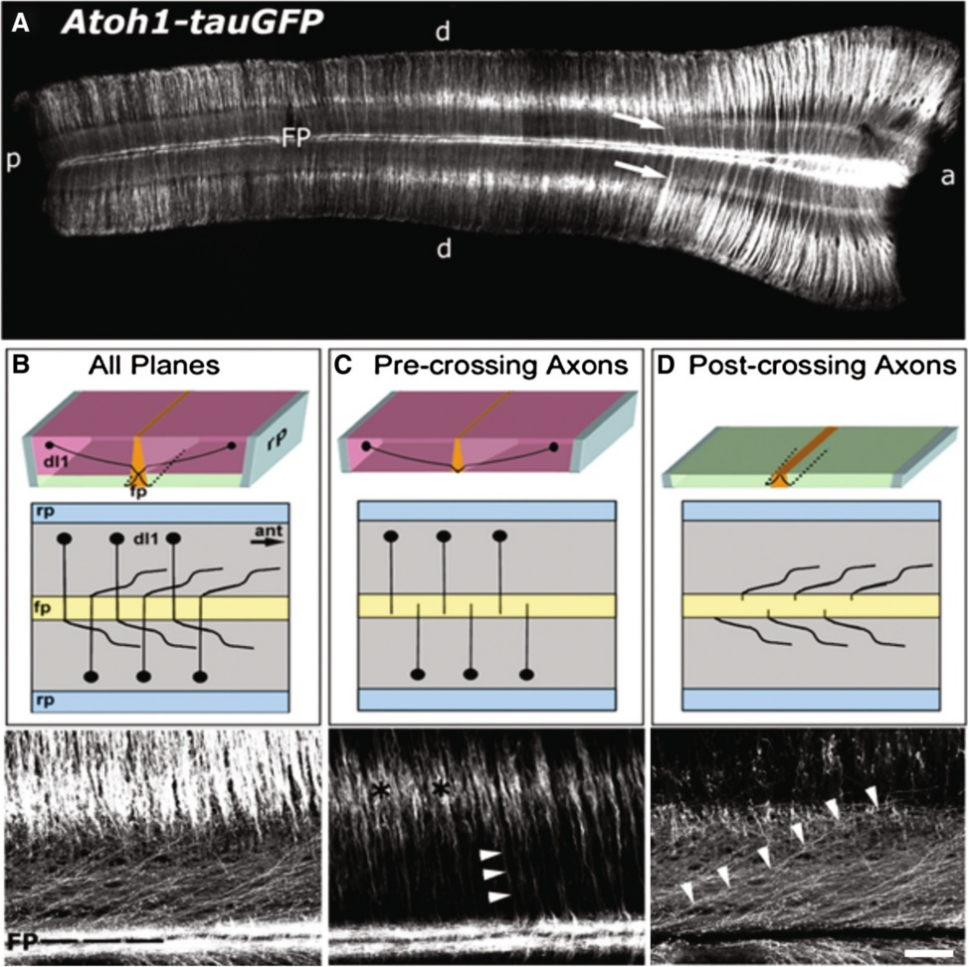
**Pre-crossing and post-crossing segments of GFP-labeled commissural axons visualized in distinct optical planes. (A)** GFP immuno-labeling of an open-book preparation spanning the thoracic (left) to cervical (right) spinal cord derived from an E11.5 *Atoh1-tauGFP* mouse. Given the bilateral symmetry of the spinal cord, commissural axons from each side cross the ventral midline and obscure the visualization of axons arising from neurons located on the opposite side. **(B)** The top panel is a three-dimensional drawing of the open-book and the middle panel is a two-dimensional schematic representing the view from above the spinal cord. The bottom panel represents a confocal micrograph (Z-stack) of all planes from the labeled open-book displayed in **A**, including pre-, midline-, and post-crossing segments of dI1 commissural axons. The labeling on each side of the FP contains post-crossing axons that project rostrally, immediately adjacent to the ventral midline. **(C)** The three- (top) and two-dimensional (middle) schematics depict the optical planes of the open-book that contain mainly dI1 commissural cell bodies and the pre-crossing axonal segments. The micrograph (bottom) is from the same region imaged in **B** that includes only optical planes containing cell bodies (black asterisks) and pre-crossing axons (arrowheads). **(D)** The three- (top) and two-dimensional (middle) schematics depict the optical planes (marginal zone of spinal cord) of the open-book that mainly contain post-crossing dI1 commissural axon segments. The micrograph (bottom) is from the same region examined in **B** and **C**, but includes only planes representing the most marginal surface of the open-book containing post-crossing axon segments (arrowheads outline one such axon). Scale bar in **D**, 500 μm for **A**, and 100 μm for **B**-**D**.

Due to the bilateral symmetry of the spinal cord, labeled dI1 (Figure 3A) and dI4 (data not shown) neurons and their axons are present on both the left and right sides of open-book preparations derived from *Atoh1-tauGFP* and *Neurog2-tauGFP* mouse embryos. Accordingly, in a given open-book preparation, pre-crossing axons on one side of the spinal cord obscure post-crossing axons originating from the opposite side and vice versa. We have previously shown that DiI-labeled pre-crossing commissural axons are located in a significantly more medial region of spinal cord open-book preparations than their post-crossing counterparts [32]. Therefore, we reasoned that it should be possible to selectively visualize pre- and post-crossing segments of dI1 and dI4 axons using confocal microscopy. By scanning through an open-book preparation derived from the spinal cord of E11.5 *Atoh1-tauGFP* (Figure 3B-D) or *Neurog2-tauGFP* (data not shown) mouse embryos from the ventricular (inner) to marginal (outer) surface, we captured planes containing predominantly pre- or post-crossing dI1 axons. Notably, as we have observed in chick embryos electroporated with the reporter constructs used to generate these mice, many post-crossing dI1 (Figure 3D) and dI4 (data not shown) axons project diagonally away from the FP. Importantly, this visualization strategy provides a means for selectively assessing the consequences of inactivating a given guidance receptor/ligand on the pathfinding of pre- and post-crossing dI1 and dI4 axons.

### The guidance of post-crossing, but not pre-crossing, dI1 axons is perturbed in Neuropilin 2 (*Npn2)* mutant spinal cords

Our observation that Npn2 is expressed on both pre- and post-crossing segments of dI1 axons (see Figure 2A) raised the possibility that this Sema3 receptor is required for these axons to navigate to, across, and/or beyond the ventral midline in the embryonic mouse spinal cord. Further, our finding that anti-Npn2 does not label most dI4 axons (see Figure 2B) suggests that Npn2 is selectively required for the guidance of dI1 axons. In order to test these possibilities, we used the visualization strategy described above to assess the consequences of inactivating Npn2 or its ligands, Sema3F/Sema3B, on the pathfinding of pre- and post-crossing dI1 and dI4 axons. Specifically, we separately crossed the *Atoh1-tauGFP* and *Neurog2-tauGFP* reporter lines with *Npn2*, *Sema3F* or *Sema3B* knockout mice and utilized confocal microscopy to selectively visualize pre- and post-crossing dI1 or dI4 axons in these mutant mice.

**Figure 4:**
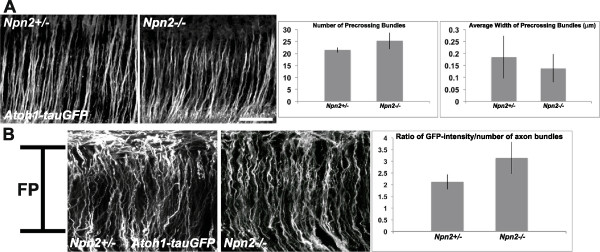
**The number of pre- and midline-crossing dI1 axons is unaltered in the absence of Neuropilin 2 (Npn2).** Confocal micrographs from Z-stack optical planes that contain exclusively pre-crossing **(A)** or midline crossing **(B)** segments of labeled dI1 axons in open-book preparations derived from E11.5 *Npn2−/+* or *Npn2−/−* mouse embryos harboring the *Atoh1-tauGFP* reporter. **(A)** The numbers of pre-crossing axon bundles were counted in images representing three separate pre-crossing axon-containing regions from the *Npn2* null or heterozygous littermate embryos. There was no statistical difference between the numbers of axon bundles in homozygous and heterozygous embryos (Student’s T-test). The width (in μm) of individual bundles was measured for the same sets of images used to calculate the numbers of axon bundles and there was no statistically significant difference (Student’s T-test) between the values obtained for *Npn2* null mouse embryos and their littermate controls. **(B)** The average GFP-intensity of each section was normalized to the number of axon bundles crossing the ventral midline at the FP to generate a ratio of GFP-intensity/axon bundles from three separate FP-containing regions of a given *Npn2* null embryo and compared with the measurements obtained from heterozygous littermate control embryos. A Student’s T-test showed no statistically significant difference between the numbers of axons in the homozygous or heterozygous preparations. The data are derived from 4 to 5 embryos per genotype, per measurement. Scale bar = 100 μm in **A**, and the width of the FP in **B** (see bar in left-most panel).

**Figure 5:**
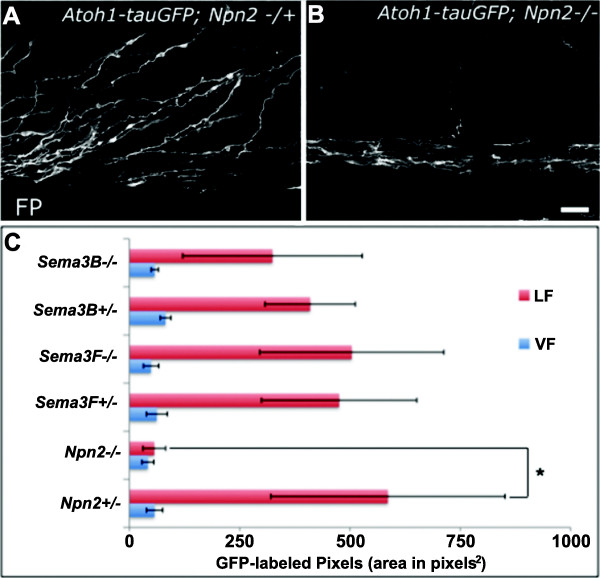
**Loss of Neuropilin 2 (Npn2) leads to a decrease number of post-crossing, *Atoh1-tauGFP-positive *spinal commissural axons. (A, B)** Confocal micrographs representing the marginal zone (post-crossing axon-containing planes) of open-book preparations derived from either *Npn2 −/+***(A)** or *Npn2 −/−***(B)** embryos harboring the *Atoh1-tauGFP* reporter. Note the paucity/absence of axons projecting away from the midline in the open-book preparation generated from the *Npn2* null embryo. **(C)** To assess the pathfinding behavior of labeled post-crossing dI1 axons in *Npn2*, *Sema3F* and *Sema3B* null mutant embryos, open-book preparations were derived from homozygous and heterozygous embryos for each line, and the samples were imaged using confocal microscopy. The outermost seven planes (0.5 mm per plane from the underside or ventral-most region of a given open-book preparation) were considered to represent the marginal zone and the areas of GFP-labeled pixels occupying the ventral funiculi (VF, defined as region from FP to 50 μm lateral to the FP) or the lateral funiculi (LF, defined as region located 50 to 200 μm lateral to the FP) were measured. GFP intensity was then averaged across all regions and the relative areas of GFP labeling were compared in homozygous and heterozygous samples. As shown in the bar graph, a statistically significant change/decrease in the area of GFP-labeled pixels, which reflects a reduced number of post-crossing dI1 axons projecting away from the FP, was only observed in the LF of *Npn2* knockout animals. The data are derived from 4 to 5 embryos per genotype. Student’s T-test , *P* <0.01. Scale bar in **B** is 25 μm for **A**-**B**.

**Figure 6:**
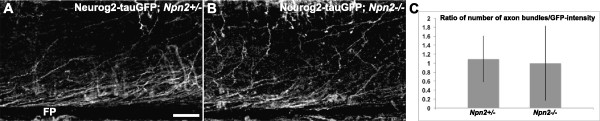
**Neuropilin 2 (Npn2) is not required for the pathfinding of post-crossing dI4 axons. (A, B)** Confocal micrographs of the post-crossing commissural axon-containing region in open-book preparations derived from the spinal cords of *Npn2−/+* and *Npn2−/−* E11.5 mouse embryos in a *Neurog2-tauGFP* reporter background. **(A)** In the *Npn2* heterozygous preparation, *Neurog2-tauGFP*-labeled dI4 axons project diagonally away from the FP, analogous to the contralateral pathfinding behavior of their dI1 counterparts (see Figure 3). **(B)** In the open-book preparation derived from a *Npn2* null littermate, post-crossing dI2 axons project diagonally away from the FP as they do in heterozygous and wild type (data not shown) embryos. **(C)** The average number of axon bundles crossing the ventral midline at the FP was normalized to the GFP-intensity of each section to generate a ratio of axon bundles/GFP-intensity. A Student’s T-test showed no statistically significant difference between the numbers of axons in the *Npn2* homozygous or heterozygous preparations. The data are derived from 4–5 embryos per genotype. Scale bar in **A**, 25 μm for **A**-**B**.

Given that dI1, but not dI4, axons express Npn2, we initially focused our analyses on the pathfinding of labeled pre-, midline- and post-crossing axons in *Atoh1-tauGFP* reporter mice lacking *Npn2*, *Sema3F* or *Sema3B*. Consistent with a lack of a role for Sema3-Npn2 signaling in regulating the guidance of dI1 axons to and across the FP, no significant alterations in the numbers or width of pre-crossing axons or the numbers of axons navigating through the FP were observed in *Npn2* (Figure 4), *Sema3F* or *Sema3B* (data not shown) null mouse embryos, as compared to their heterozygous littermate controls. In striking contrast, most post-crossing dI1 axons fail to project away from the FP along wild type-like diagonal trajectories in *Npn2* null embryos as compared to heterozygous littermate controls (Figure 5A, B). Notably, however, qualitative analyses failed to identify contralateral dI1 projection defects in mice lacking *Sema3F* or *Sema3B* (data not shown). To obtain a measure of the relative numbers of post-crossing commissural axons that projected away from the FP along diagonal trajectories within the lateral funiculus (LF), confocal images obtained from each open-book preparation were used to generate YZ projections. Subsequently, Metamorph software was used to select single YZ planes at 100-plane intervals along the X-axis and to quantify the area occupied by GFP-labeled axons within the LF. The LF was defined as the region located between 34 and 200 μm lateral to the FP. These analyses confirmed the relative and selective absence of diagonally projecting dI1 axons in *Npn2* null embryos, as compared to mice lacking Sema3F or Sema3B (Figure 5C). In contrast to these observations, but consistent with the lack of Npn2 expression on most dI4 axons, pre-, midline- and post-crossing GFP-positive commissural axons from *Neurog2-tauGFP* embryos project in a wild type-like manner in mice lacking *Npn2* (post-crossing, Figure 6; pre-crossing, data not shown) or *Sema3F/Sema3B* (data not shown). Together, these observations suggest that Npn2 is selectively required for guiding post-crossing dI1 axons away from the ventral midline.

### Post-crossing dI1, but not dI4, axon-associated growth cones collapse in the presence of *Sema3B, Sema3F or Slit2*, *in vitro*

**Figure 7:**
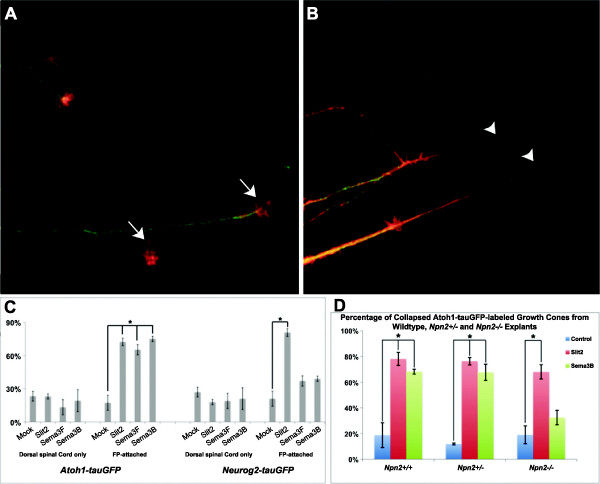
**Sema3B or Sema3F collapses growth cones associated with post-crossing dI1, but not dI4, axons.** Floor plate (FP)-attached (post-crossing) and dorsal spinal cord only (pre-crossing) explants containing dorsal commissural neurons were generated from open-book preparations of E11-E11.5 *Atoh1-tauGFP***(A, B)** or *Neurog2-tauGFP* (data not shown) spinal cords. **(A)** Growth cones (GCs) from a post-crossing explant derived from an *Atoh1-tauGFP* open-book and labeled with Phalloidin (red) and GFP (green) remained well spread after 1 h mock-conditioned medium treatment. **(B)** Many of these GCs collapsed (absence of lamellipodia/filopodia) when treated for 1 h in media conditioned with Slit2, Sema3F or Sema3B. **(C)** The percentage of collapsed GCs associated with *Atoh1-tauGFP* (dI1) or *Neurog2-tauGFP* (dI4) pre-crossing axons treated with Slits-, Sema3F- or Sema3B-conditioned media was not significantly different as compared to mock. A significant increase in the percentage of collapsed GCs was observed with post-crossing dI1 axons treated Slit2, Sema3F or Sema3B, as compared to mock-media (chi-squared test, *P* value <0.05). No difference in the percentage of collapsed GCs was observed with post-crossing dI4 axons in the presence of Sema3B or Sema3F, as compared to mock-media. A significant increase in the collapse of dI4 axon-associated GCs was observed when treated with Slit2 as compared to mock-media (chi-squared test, *P value <0.05). **(D)** Growth cones from *Npn2*^*+/+*^ and *Npn2*^*+/−*^ post-crossing axons displayed significant increases (chi-squared test, *P value <0.05) in the percentage of collapse when treated with Slit2 or Sema3B, as compared to mock-media. Sema3B failed to induce a significant increase over mock in the percentage of collapsed GCs from *Npn2*^*−/−*^ embryos. The data are derived from 4 to 5 embryos/genotype. The percentage of collapse was calculated as the number of collapsed GCs divided by the total number of GCs. ±50 GCs were analyzed for each condition and in each experiment.

To provide further support for the notion that Npn2 selectively regulates the contralateral pathfinding of dI1 axons, we carried out growth cone collapse assays (see [33-35]) to individually assess the responsiveness of pre- and post-crossing dI1 and dI4 axon growth cones to the midline repellents, Sema3B, Sema3F and Slit2. Specifically, we grew FP-lacking, dorsal spinal cord only (source of dorsal spinal neuron cell bodies and their pre-crossing axons/growth cones) or FP-attached half spinal cord (source of dorsal spinal neuron cell bodies and their post-crossing axons/growth cones) explants derived from E11.5 *Atoh1-tauGFP* or *Neurog2-tauGFP* mouse embryos on cover slips (see Methods) until the axons extending from these explants had elaborated well-spread growth cones. Subsequently, Sema3B, Sema3F or Slit2 conditioned media (generated as described in Methods) was applied to the explants for 1 hour and then the explants were fixed, permeabilized and labeled with AlexaFluor-568 conjugated Phalloidin (Molecular Probes; see Methods). The morphology of dI1 and dI4 growth cones was then scored for collapse by epifluorescence microscopy based on the well-established criteria that collapsed growth cones lack lamellopodia and multiple filopodia [35]. Consistent with the expression studies and knockout mouse analyses described above, Sema3F and Sema3B promote the collapse of a significant percentage of dI1, but not dI4, growth cones, with only post-crossing (not pre-crossing) dI1 axon-associated growth cones displaying responsiveness to Sema3 conditioned medium (Figure 7A-B). Supporting a role for Npn2 in facilitating this inhibitory response, Sema3B failed to promote a significant increase in the percentage of collapsed post-crossing dI1 axon-associated growth cones lacking Npn2 (Figure 7D). Given the likely expression of repulsive Slit receptors, Robos, on post-, but not pre-, crossing dI1 and dI4 axons (see [16,31]), Slit2 conditioned medium promoted the selective collapse of post-crossing dI1 and dI4 axons (Figure 7C). Consistent with Robos, but not Npn2, being required for responsiveness to Slits, Slit2 also collapsed dI1 axon-associated growth cones lacking Npn2 (Figure 7D).

### Ventral spinal commissural neurons and their axons express GAD65

**Figure 8:**
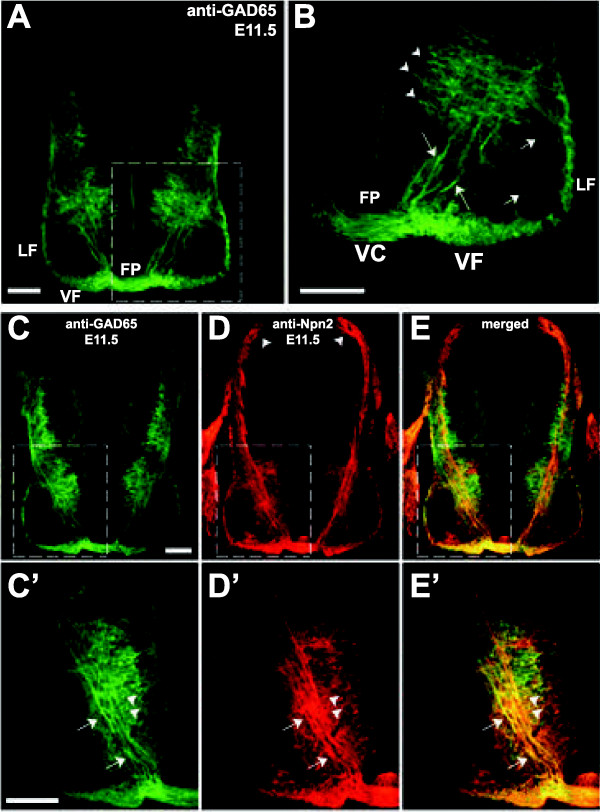
**Ventral spinal commissural neurons are GABAergic and express Neuropilin 2 (Npn2). (A-B)**, A wild type E11.5 mouse spinal cord transverse section is labeled with anti-GAD65 to visualize the large population of GABAergic commissural neurons (CNs) with cell bodies (arrowheads) located in the ventromedial region of the intermediate zone (dashed box area is shown at high magnification in **B**). Axons (arrows in **B**) emanating from ventromedial GABAergic CNs project ventrally toward the floor plate (FP) and cross the midline within the ventral commissure (VC), and then turn rostrally to join the ventral funiculus (VF). Some GAD65-positive neurons also extend their axons ipsilaterally (shorter arrows in **B**) to join the lateral funiculus (LF). **(C-E’)**, Some GAD65-positive CNs from the ventromedial population also express Npn2 on the ipsi- and contralateral segments of their axons as illustrated by the double immunofluorescence (**E**, merge) labeling of a single spinal cord transverse section with anti-GAD65 (**C**, green) and anti-Npn2 (**D**, red). Higher magnification of the dashed boxed areas in the single **(C-D)** or double/merge **(E)** labeled section are shown in **C’-E’**, and reveal that a subset of the ventromedial GABAergic CNs expresses both GAD65 and Npn2 on their axons (arrows) and cell bodies (arrowheads). Scale bars in **A** and **C**, 50 μm for **A**, **C**-**E**; in **B** and **C’**, 100 μm for **B**, **C’**-**E’**.

Previous studies in rat embryos utilizing an antibody specific for rat GAD65, one of the two rate-limiting enzymes required for the synthesis of GABA, identified a novel population of GABAergic commissural neurons, with cell bodies located in the ventromedial spinal cord [17,18]. These GABAergic commissural neurons can be visualized as early as E9.5 and their axons grow ventrally toward the FP, cross the midline and project orthogonally, within ventral and lateral funiculi by E12.5 in the embryonic rat spinal cord [17]. In contrast to dorsal commissural neurons (see [15]), a subset of these GABAergic commissural neurons were shown to express the cell adhesion molecule L1 on both the pre- and post-crossing segments of their axons. Ultimately, the GAD65/L1-expressing post-crossing axons project rostrally within the ventral funiculi to midbrain targets [36,37]. To determine whether GABAergic commissural neurons also exist in ventral regions of the embryonic mouse spinal cord we examined the labeling pattern of an antibody specific for mouse GAD65. Anti-mouse GAD65 labels a large population of GABAergic neurons located in a ventromedial region of the mouse spinal cord at E11.5 (Figure 8). Interestingly, some of the GAD65-positive neurons with cell bodies located more dorsally within the ventromedial population extend their axons ipsilaterally toward the lateral funiculus, supporting the view that these GABAergic spinal neurons do not represent a pure commissural population. In addition to the larger ventromedial population, a smaller GAD65-positive population with cell bodies located dorsolaterally to the ventromedial neurons was also observed to extend their axons ventrally toward the FP and cross the ventral midline (Figure 8). As in the rat, we also observed a smaller population of GAD65-positive cell bodies located dorsal to GABAergic commissural neurons at this stage that most likely represents GABAergic interneurons, which reside in the deep dorsal region of the spinal cord. Collectively, the distribution of GAD65-positive neuronal cell bodies and the projections of their axons suggest that they are the mouse counterparts of the GABAergic embryonic spinal rat neurons/axons that we have previously described (see references above).

### Neuropilin 2 is required for the guidance of post-, but not pre-, crossing GABAergic ventral commissural axons

**Figure 9:**
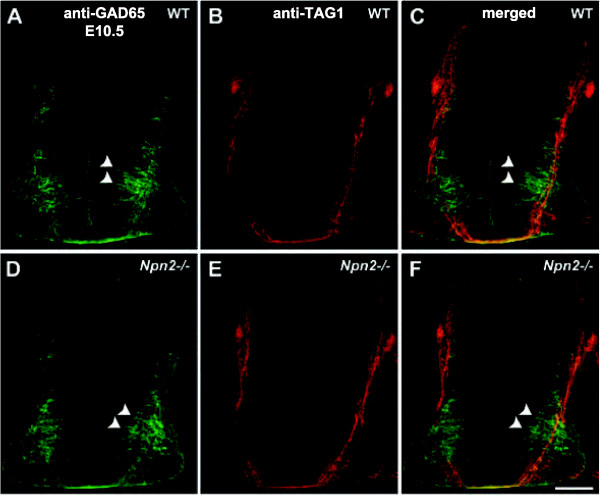
**GABAergic spinal commissural axon trajectory is normal in the E10.5 *Npn2***^***−/−***^**spinal cord. (A-C)**, A wild type E10.5 transverse spinal cord section at the cervical level is double-labeled with anti-GAD65 (**A**, green) and anti-TAG1 (**B**, red), or merged of the same section **(C)**. At this developmental stage, GABAergic commissural neurons in the ventromedial spinal cord can be prominently labeled and a few axons have projected across the floor plate to the contralateral side. **(D-F)**, An *Npn2*^*−/−*^ E10.5 cervical spinal cord section from the same litter is double-labeled with anti-GAD65 (**D**, green) and anti-TAG1 (**E**, red), or merged of the same section **(F)**. Both GAD65- and TAG1-positive commissural axons are seen to project across the ventral midline in the *Npn2*^*−/−*^ spinal cord. Scale bar in **F**, 25 μm for **A**-**F**.

**Figure 10:**
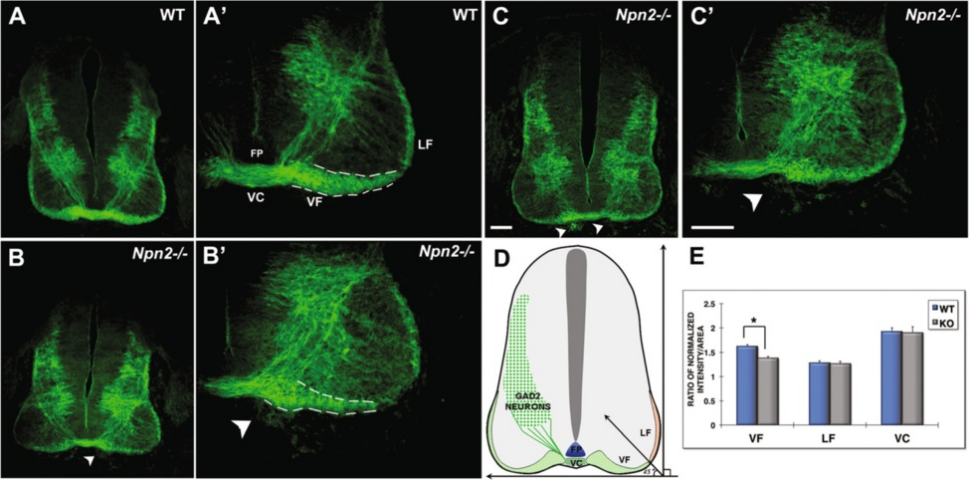
**Neuropilin 2 (Npn2) appears to be minimally required for the contralateral pathfinding of GABAergic spinal commissural axons. (A-C’)**, Anti-GAD65 immunohistochemistry was used to assess the development of GABAergic commissural neurons and their pre- and post-crossing axonal segments in transverse sections derived from *Npn2*^*−/−*^ mouse embryos and wild type littermates. A rather modest thinning of the ventral funiculus (VF) was observed in *Npn2*^*−/−*^**(B**, **B’**, **C**, **C’)**, as compared to WT **(A**, **A’)** littermate control spinal cords. Consistent with labeled post-crossing axons destined for the VF following mis-guided trajectories in the absence of Npn2, we identified aberrant GAD65-positive fibers (arrowheads) outside of the spinal cord at the edges of the ventral commissure (VC) or immediately ventral to the VF. **(D)**, A schematic diagram indicating the positions of GABAergic neurons and the ventral (VF) and lateral (LF) funiculi used for quantification in E. **(E)**, Quantification of the normalized fluorescence intensity with a given domain revealed fewer number of axons in the VF, but no significant differences in the width of the ventral commissure (VC) or LF. The ratio of the normalized intensity/area was calculated by averaging the normalized fluorescent intensity (intensity of the specific region/total intensity of the section) over the normalized area (area of the specific region/entire area of the spinal cord). Student’s t-test, *P <0.01; n =4 to 5 embryos per genotype, ten 25 μm thick sections per embryos were analyzed. Scale bars in **C**, 40 μm for **A**-**C**; in **C’**, 80 μm for **A’**-**C’**.

To determine whether GABAergic ventral commissural axons require class 3 Sema signaling for their guidance, just as their dorsal counterparts do, we first asked whether ventral commissural neurons/axons express Npn2. Using an antibody specific for Npn2, we observed that many ventromedially located GAD65-positive commissural neurons and both their pre- and post-crossing axons express Npn2 (Figure 8), however, not all GABAergic commissural axons are anti-Npn2 positive. Notably, the smaller, dorsolaterally located population of GAD65-positive commissural neurons appears to be Npn2-negative. To determine whether those GAD65-positive commissural axons that express Npn2 on their axons require Sema3 signaling for their axon guidance at the midline or pathfinding within the ventral funiculus, we examined these axons at a stage when the majority are beginning to cross, or project across, the ventral midline from E10.5 to E11.5 in transverse sections derived from *Npn2* homozygous mice (Figures 9 and 10). No gross midline guidance defects were displayed by the GAD65-positive axons at E10.5 or E11.5 in the cervical and thoracic spinal cord (Figures 9 and 10). Both the pre- and midline- (within the ventral commissure) crossing segments of GABAergic commissural axons elaborate wild type-like projections in the *Npn2*^*−/−*^ animals, as compared to age-matched littermate controls. In contrast, a relatively minor defasciculation (or disorganization) and thinning of GAD65-positive post-crossing axons projecting within the ventral funiculus was detected at E11.5 (Figure 10).

## Discussion

### Labeling strategies for visualizing discrete populations of commissural neurons in the developing mouse spinal cord

By exploiting unilateral *in ovo* electroporation in the embryonic chick spinal cord, we previously showed that *Atoh1* and *Neurog1* enhancer elements direct reporter expression to distinct classes of dorsal commissural and characterized the trajectories of their pre- and post-crossing axons [16,31]. Here, we used enhancer elements to generate *Atoh1-tauGFP* and *Neurog2-tauGFP* reporter mice and to visualize the trajectories adopted by dI1 and dI4 commissural axons, respectively, in the embryonic mouse spinal cord. Although pre-crossing dI4 axons projected to the FP along a more lateral route than their dI1 counterparts, confocal microscopy facilitated the optical separation of planes containing pre- and post-crossing axon segments, revealing that a large number of both dI1 and dI4 post-crossing axons extend away from the FP along diagonal trajectories. Supporting the validity of these observations, the post-crossing projections of mouse dI1 axonal subtypes closely resembled those displayed by their chick counterparts [16]. We also used an anti-mouse GAD65 antibody to identify, for the first time, a ventral population of commissural neurons that extends post-crossing axons into the both the ventral and lateral funiculus of the mouse spinal cord. These particular findings are consistent with our previous observations in the embryonic rat spinal cord [17,18,36,37].

In principle, the labeling strategies that we describe here should make it possible to assess and compare the consequences of inactivating any receptor-ligand system or other factor(s) on the guidance of dorsal and ventral commissural axons in the embryonic mouse spinal cord. The roles of a given guidance molecule in directing the growth of dorsal and ventral commissural axons could be investigated in the same embryos by labeling spinal cord preparations derived from dI1 or dI4 (dorsal populations) reporter mice, which have been labeled with anti GAD65 (ventral population) and crossed with mice deficient in the candidate gene. Alternatively, dI1 or dI4 reporter mice could be mated with GAD65 reporter animals (see [38]) - with a dorsal reporter line harboring a GFP reporter and the GAD65 line carrying a mCherry reporter, or vice versa - and the dual-labeled mice crossed with a knockout line of interest. In addition to evaluating the consequences of inactivating putative guidance factors on the pathfinding of dorsal and ventral commissural neurons within the spinal cord proper, it should also be possible to assess the effects of these perturbations on the long-distance projections of these axons to their brain targets utilizing the labeling strategies described herein. Alternatively, these particular analyses could be carried out by delivering dI1, dI4 and GAD65 reporter constructs into the embryonic mouse spinal cord via unilateral *in utero* electroporation (see [31,39]). Based on our previous findings in chick [31] and rat [37] embryos, we expect that at least a subset of mouse dI1 and dI4 commissural axons will project within spinocerebellar tracts, whereas a significant number of GAD65-positive mouse GABAergic commissural axons will assemble into spinomesencephalic tracts. Given that the GAD65 neurons we describe in this study are born slightly before the neurons that compose most spinal ascending axon tracts (see [40]), we further suggest that the GAD65-positive neurons, in particular, likely represent pioneer neurons, which contribute axons to some of these tracts.

### Compensatory roles of Sema3B and Sema3F in guiding post-crossing Atoh1 (dI1) commissural axons

Using Atoh1-tauGFP reporter mice, we show that dI1 post-crossing commissural axons require Npn2 for their contralateral trajectory away from the floor plate. In addition, we show that both Sema3B and Sema3F promote robust collapse of post-crossing dI1 axon-associated growth cones. However, neither the Sema3B nor the Sema3F single knockouts phenocopy the *Npn2* mutant mice. Thus, our results raise the possibility that Sema3B and Sema3F have compensatory or redundant roles in mediating post-crossing Npn2-positive commissural axon guidance. Interestingly, Slit ligands have been shown to operate in a collaborative manner to regulate midline crossing of commissural axons in both the spinal cord and retina [20,22,41]. To determine whether Sema3B and Sema3F have redundant roles in driving post-crossing dI1 projections away from the FP we would need to assess axon pathfinding in Sema3B and Sema3F double knockout spinal cords. However, the close association between the Sema3B and Sema3F loci, which are located less than 0.072 mega base pairs away from each other on the same chromosome, precludes the execution of these experiments. Thus, additional experiments, beyond the scope of this study, are required to definitively determine whether Sema3B and Sema3F operate in concert to guide post-crossing Npn2-positive axons away from the FP.

### Neuropilin 2 selectively regulates the guidance of a subset of post-crossing commissural axons

Utilizing dI1 and dI4 reporter mice and anti-GAD65 immunohistochemistry we assess here, for the first time, the consequences of inactivating Npn2 on the pathfinding of distinct subsets of dorsal and ventral commissural axons. Consistent with our observation that Npn2 is expressed on dI1 and GAD65-positive, but not dI4, commissural axons, we show that Npn2 is selectively required for the contralateral pathfinding of dI1 and GABAergic ventral commissural axons. Notably, the inactivation of Npn2 more profoundly disrupts the guidance of dorsal as opposed to ventral commissural axons; in *Npn2* null embryos most post-crossing dI1 axons fail to project away from the FP along diagonal trajectories, whereas GAD65-postiive post-crossing exhibit rather minor de-fasciculation defects within the ventral funiculus. It is interesting to note in this regard that the midline attractant, Netrin-1, has been shown to preferentially guide dorsal as opposed to ventral spinal commissural axons [42]. Despite the fact that post-crossing dI4 axons also project away from the FP along diagonal trajectories, which are similar in shape to post-crossing dI1 projections, these axons pathfind normally in mice lacking Npn2. As indicated above, this is consistent with our finding that dI4 neurons/axons do not express Npn2. Interestingly, Robo2 is required for driving most dorsal post-crossing axons away from the ventral midline [22]. Accordingly, it is possible that Robo2 has a major role in directing Npn2-negative dI4 commissural axons away from the FP along diagonal trajectories. Together, our observations support the view that Npn2 regulates commissural axon guidance in a population-specific manner and raise the possibility that *Atoh1* or another transcription factor expressed by post-mitotic dI1 neurons directly regulates the expression of Npn2. However, our preliminary studies indicate that Npn2 expression is unaltered in mice lacking Atoh1 (EC and ZK, unpublished observation) and Npn2 was not identified in a systematic screen for direct lineage-specific *in vivo* targets of *Atoh1*[43].

We have previously shown that as a consequence of disabling Slit-Robo signaling in chick or mouse embryos post-crossing dI1 and dI2 axons fail to project diagonally away from the FP [16,31]. These phenotypes closely resemble the contralateral pathfinding defects displayed by dI1 axons in mice lacking Npn2, raising the possibility that Robo and Npn receptors functionally and/or physically interact to regulate commissural axon pathfinding. Notably, Robo-Npn2 interactions facilitate cortical interneuron migration within the embryonic mouse forebrain [44]. Alternatively, repulsive Robo receptors and Npn2 may separately regulate the guidance of heretofore-unidentified subsets of dI1 axons. We do not favor this possibility since the dI1 axons disrupted as a result of disabling Robo signaling in electroporated chick embryos, and those perturbed following inactivation of Npn2 in transgenic reporter mouse embryos, were visualized utilizing the same *Atoh1* enhancer elements. Given that dI1 axons likely contribute to the spinocerebellar tract and GABAergic ventral commissural axons presumably assemble into spinomesencephalic tracts (see above), it would be interesting to determine, in future studies, whether inactivation of Npn2 disrupts the formation of these longitudinally projecting ascending axonal tracts. If the loss of Npn2 perturbs dI1 axons from forming a spinocerebellar tract in mouse embryos, this would be consistent with the targeting phenotype we observed in chick embryos following the disruption of Slit-Robo signaling [31], and represent another functional parallel between the roles of Robo and Npn receptors in commissural axon pathfinding.

Consistent with commissural axons gaining responsiveness to midline repellents only after they cross the FP, it was previously shown that the growth of cultured post-, but not pre-, crossing commissural axons are responsive to midline-associated Sema3s [26]. Complementing and extending this observation by identifying a subset of dorsal commissural axons that gain responsiveness to midline inhibitory cues, we show here that Sema3B/3F selectively promotes the collapse of post-crossing dI1 (but not dI4) commissural axon-associated growth cones. Given that we find Npn2 expressed on both pre- and post-crossing segments of dI1 axons it is not clear why growth cones associated with post-crossing axons are selectively responsive to midline Semas, but the underlying mechanism could involve axon segment-specific receptor processing [27,29] or silencing [45]. It is well established that Npns form holoreceptor complexes with class A Plexins in order to mediate repulsion [25,46,47]. Accordingly, commissural axon-associated PlexinA1 [27] is a good candidate for facilitating the response of post-crossing dI1 growth cones to Sema3B/3F and this possibility can be addressed by analyzing the consequences of inactivating Plexin A1 in *Atoh1-tauGFP* reporter mice.

## Conclusions

Numerous molecularly distinct subsets of commissural neurons are distributed throughout the vertebrate spinal cord (see Background). Despite this well-established diversity, whether the same set of guidance receptors-ligands controls the pathfinding of all midline-crossing commissural axon populations or whether the directed growth of each population is regulated by a particular subset of these guidance systems, as has elegantly been shown for spinal motor axons [48,49], remains a key open question in the field. In large part, the lack of robust and reproducible labeling systems that can be used to reliably visualize, and assess the consequences of a given perturbation on, distinct classes of commissural axons has precluded population-specific analyses of commissural axon pathfinding. Here, we utilize novel genetic labeling strategies and immunohistochemistry to elucidate the distinct axonal trajectories of genetically specific populations of commissural neurons along the dorsoventral spinal cord. In addition, we show that the dI1, not the dI4, population of commissural neurons derived from *Atoh1* progenitors, and a subset of the GABAergic ventral commissural neurons express the Npn2 receptor on all segments of their axons. However, only the contralateral/post-crossing segments of dI1, Atoh1-GFP labeled axons respond to Sema3-mediated repulsion. Taken together our findings show that Sema3-Npn2 signaling is required for the pathfinding of distinct subtypes of contralateral commissural axons in the developing mouse spinal cord.

## Methods

### Mice

All mice were maintained on a C57BL/6 background and at least three backcrosses were performed for each line. For timed pregnancies, embryonic day 0.5 (E0.5) was considered to be noon on the day the vaginal plug was observed. The *Neurog2-tauGFP* transgenic mice were generated using the TgN2-7tauGFP transgene according to standard procedures by the University of Texas Southwestern Medical Center Transgenic Facility. The transgene contains a 1 kb enhancer (chr3:127337663 to 127338713 from mouse build mm9) from the 3’ end of the *Neurog2* gene that has been mutated such that, in combination with the β-globin basal promoter, it directs reporter expression to a subset of *Neurog2*-expressing progenitors within the dorsal neural tube in transgenic mice [12]. The tauGFP cassette is as published for *Atoh1tauGFP*[15]. The *Npn2* and *Sema3F* mutant mice were maintained as previously described [50]. The *Sema3B* mutant mouse line was purchased from the Jackson Laboratory (strain #006705) and maintained according to the instructions on their website. In all cases, genotyping was performed using the PCR and DNA samples generated from mouse ear or tail tissue biopsies. Pregnant dams were exposed to compressed carbon dioxide and sacrificed by cervical dislocation and the embryos were removed by cesarean section. The Institutional Animal Care and Use Committees of the Albert Einstein College of Medicine and Rutgers University collectively approved the animal-use protocols.

### Immunohistochemistry

Timed-pregnant mice were sacrificed at a given embryonic day and the embryos were harvested in cold PBS. Embryos were then immersion-fixed in cold 4% Paraformaldehyde for 2 to 4 h and equilibrated in a 30% sucrose solution overnight, before being embedded in Tissue-Tek OCT compound (Miles Scientific, Elkhart, IN, USA) and frozen at −80°C. Embryos were cryosectioned at 16 or 20 μm using a Leica Cryostat and the sections were mounted on Superfrost Plus microscope slides (Fisher Scientific International) and allowed to air dry for 16 h at room temperature.

Immunohistochemical labeling of the sections was performed essentially as described [51,52]. A goat polyclonal antibody specific for Npn2 (cat. no. AF567; R&D Systems, Minneapolis, MN, USA) was used at 15 μg/ml, and a rabbit monoclonal anti-GAD2/GAD65 was used at 1:1000 (cat. no. 5843, Cell Signaling, Danvers, MA, USA) was used at 1:1000, with each antibody diluted in 10% donkey serum and 0.1% Triton X-100 and applied to the sections after blocking in the same buffer. Appropriate secondary antibodies were obtained from Jackson ImmunoResearch Laboratories, Inc (West Grove, PA, USA).

### Analyses of open-book spinal cord preparations

Open-book preparations of embryonic spinal cords were generated from the various reporter mice in a given genetic background as described [34]. To visualize the projections of the resident GFP-labeled axons, the open-book preparations were flat-mounted and imaged using an Olympus Fluoview 500 confocal microscope. Image stacks/Z series were analyzed using Metamorph Imaging Software (Universal Imaging, Inc.). To analyze the relative density of GFP-labeled axons contained within a particular portion of these preparations, the images were set to threshold using the auto-threshold function and the number of GFP-positive pixels was counted. Comparable levels of the rostral-caudal axis of the spinal cord were analyzed in each set of experiments, and the thresholding of the images was kept consistent between all image sets. Data was represented as an average of the area containing fluorescence over the threshold value. Data sets between mutant and control groups were then compared using a Student’s-T test.

### *In vitro* collapse assay

Large open-book preparations were dissected from E11.5 mouse embryos of various genotypes in ice-cold, Dulbecco's modified Eagle's medium (DMEM; Gibco-BRL, Carlsbad, CA, USA) according to previous published methods [33,34]. For pre-crossing axon containing explants, tungsten needles were used to isolate floor plate-lacking dorsal spinal cord tissue, which contains cell bodies of commissural neurons. In contrast, we isolated floor-plate-attached commissural neuron containing half spinal cord explants as sources of post-crossing axons. Dorsal spinal cord preparations (pre-crossing axons) were supplemented with 250 ng/ml recombinant Netrin-1 (cat. no. 1109-N1-025; R&D Systems, Minneapolis, MN, USA) to promote axon outgrowth. Both dorsal spinal cord (pre-crossing axons) and FP-attached preparations (post-crossing axons) were sectioned into small, approximately square, pieces and these explants were placed at 37°C with minimal media on nitric acid cleaned, Silanized, and Laminin-coated cover slips (mouse, Invitrogen cat# 23017, Carlsbad, CA, USA) in Hanks Balanced Salt Solution (HBSS, Gibco, cat#14170-088, Carlsbad, CA, USA), as described [35] for at least 2 h to allow adequate time for the tissues to adhere to the cover slips. The explants were then cultured for either 48 h in DMEM media with 1% penicillin/streptomycin/glutamine (Gibco-BRL, Carlsbad, CA, USA), and 1% Bottenstein's N2 supplement (Gibco-BRL, Carlsbad, CA, USA). To induce collapse, media was collected from HEK-293 cells transfected [32] with mammalian expression vectors containing the coding regions of Slit-2 (gift from Y Rao, National Institute of Biological Sciences) or Sema3F/Sema3B (gifts from A Kolodkin, The Johns Hopkins University). These conditioned media or control medium (from mock-transfected cells) were added to the explant cultures at a dilution of 1:100, and the explants were incubated for an additional hour at 37°C. The culture media was then removed and the explants were fixed for 10 m in pre-warmed 4% PFA with 10% sucrose. After fixation, the explants were washed with PBS and stained with AlexaFluor 568-phalloidin (cat. no. A12380; Molecular Probes, Eugene, OR, USA) and anti-GFP, AlexaFluor 488 conjugate (cat. No. A-21311; Molecular Probes, Eugene, OR, USA). The tips of axons displaying prominently spread growth cones containing lamellipodia and multiple filopodia (visualized by Phalloidin labeling) were scored as non-collapsed, whereas those lacking lamellipodia and multiple filopodia were scored as collapsed.

### Photodocumentation and data analyses

All epifluorescence images were captured using a Nikon Eclipse TE300 microscope (Nikon, Tokyo, Japan) and all confocal images were obtained with either a Fluoview 500 microscope (Olympus, Tokyo, Japan) or a Yukogawa CSU10 confocal system, and processed with ImageJ64 (National Institutes of Health, Bethesda, MD, USA). Brightness and contrast of images were adjusted using Adobe Photoshop CS (Adobe, San Jose, CA, USA). All data analyses were carried out using the statistical tests indicated in the Figure Legends and GraphPad Prism (Version 5.0d).

## Abbreviations

bHLH: basic-helix-loop-helix; CA: Commissural axon; CN: Commissural neuron; CNS: Central nervous system; dI: dorsal interneuron; D-V: Dorsoventral; FP: Floor plate; M-L: Mediolateral; LF: Lateral funiculus; Npn2: Neuropilin 2; Sema3: Class 3 semaphorin; VF: Ventral funiculus; VC: Ventral commissure.

## Competing interests

All authors declare that they have no competing interests.

## Authors’ contributions

EC and TST performed the majority of the experiments, collected, and analyzed the data, while RL and EM did the GAD65 measurements. JEJ made the *Atoh1-tauGFP* and *Neurog2-tauGFP* reporter lines. TST and ZK designed the experiments, analyzed the results and wrote the paper. All authors read and approved the final manuscript.

## Authors’ information

Tracy S Tran and Edward Carlin are co-authors.

## References

[B1] Bernhardt R Chitnis A Lindamer L Kuwada J Identification of spinal cord neurons in the embryonic and larval zebrafish J Comp Neurol 1990 302 607 616 10.1002/cne.903020315 1702120

[B2] Silos-Santiago I Snider WD Development of commissural neurons in the embryonic rat spinal cord J Comp Neurol 1992 325 514 526 10.1002/cne.903250405 1469113

[B3] Roberts A Early functional organization of spinal neurons in developing lower vertebrates Br Res Bull 2000 53 585 593 10.1016/S0361-9230(00)00392-0 11165794

[B4] Stokke M Nissen U Glover J Kiehn O Projection patterns of commissural interneurons in the lumbar spinal cord of the neonatal rat J Comp Neurol 2002 447 349 359 11954034 10.1002/cne.10211

[B5] Helms A Johnson J Specification of dorsal spinal cord interneurons Curr Opin Neurobiol 2003 13 14 49 10.1016/s0959-4388(03)00010-2 12593981

[B6] Kadison SR Kaprielian Z Diversity of contralateral commissural projections in the embryonic rodent spinal cord J Comp Neurol 2004 472 411 422 10.1002/cne.20086 15065116

[B7] Helms AW Johnson JE Progenitors of dorsal commissural interneurons are defined by MATH1 expression Development 1998 125 919 928 9449674 10.1242/dev.125.5.919

[B8] Gowan K Helms AW Hunsaker TL Collisson T Ebert PJ Odom R Johnson JE Crossinhibitory activities of Ngn1 and Math1 allow specification of distinct dorsal interneurons Neuron 2001 31 219 232 10.1016/S0896-6273(01)00367-1 11502254

[B9] Helms AW Battiste J Henke RM Nakada Y Simplicio N Guillemot F Johnson JE Sequential roles for Mash1 and Ngn2 in the generation of dorsal spinal cord interneurons Development 2005 132 2709 2719 10.1242/dev.01859 15901662 PMC1351036

[B10] Helms AW Abney A Ben-Arie N Zoghbi H Johnson JE Autoregulation and multiple enhancers control Math1 expression in the developing nervous system Development 2000 127 1185 1196 10683172 10.1242/dev.127.6.1185

[B11] Lumpkin EA Collisson T Parab P Omer-Abdalla A Haeberle H Chen P Doetzlhofer A White P Groves A Segil N Johnson JE Math1-driven GFP expression in the developing nervous system of transgenic mice Gene Expr Patterns 2003 3 389 395 10.1016/S1567-133X(03)00089-9 12915300

[B12] Simmons A Horton S Abney A Johnson JE Neurogenin2 expression in ventral and dorsal spinal neural tube progenitor cells is regulated by distinct enhancers Dev Biol 2001 229 327 329 10.1006/dbio.2000.9984 11203697

[B13] Henke RM Savage TK Meredith DM Glasgow SM Hori K Dumas J MacDonald RJ Johnson JE Neurog2 is a direct downstream target of the Ptf1a-Rbpj transcription complex in dorsal spinal cord Development 2009 136 2945 2954 10.1242/dev.035352 19641016 PMC2723066

[B14] Nakada Y Parab P Simmons A Omer-Abdalla A Johnson JE Separable enhancer sequences regulate the expression of the neural bHLH transcription factor neurogenin 1 Dev Biol 2004 271 479 487 10.1016/j.ydbio.2004.04.021 15223348

[B15] Imondi R Jevince A Helms AW Johnson JE Kaprielian Z Mis-expression of L1 on pre-crossing spinal commissural axons disrupts pathfinding at the ventral midline Mol Cell Neurosci 2007 36 462 471 10.1016/j.mcn.2007.08.003 17884558 PMC2111042

[B16] Reeber SL Sakai N Nakada Y Dumas J Dobrenis K Johnson JE Kaprielian Z Manipulating Robo expression in vivo perturbs commissural axon pathfinding in the chick spinal cord J Neurosci 2008 28 8698 8708 10.1523/JNEUROSCI.1479-08.2008 18753371 PMC2886497

[B17] Phelps PE Alijani A Tran TS Ventrally located commissural neurons express the GABAergic phenotype in the developing rat spinal cord J Comp Neurol 1999 409 285 298 10.1002/(SICI)1096-9861(19990628)409:2<285::AID-CNE9>3.0.CO;2-7 10379921

[B18] Tran TS Alijani A Phelps PE Unique developmental patterns of GABAergic neurons in rat spinal cord J Comp Neurol 2003 456 112 126 10.1002/cne.10511 12509869

[B19] Dickson BJ Gilestro GF Regulation of commissural axon pathfinding by Slit and its Robo receptors Annu Rev Cell Dev Biol 2006 22 651 675 10.1146/annurev.cellbio.21.090704.151234 17029581

[B20] Long H Sabatier C Ma L Plump A Yuan W Ornitz DM Tamada A Murakami F Goodman CS Tessier-Lavigne M Conserved roles for Slit and Robo proteins in midline commissural axon guidance Neuron 2004 42 213 223 10.1016/S0896-6273(04)00179-5 15091338

[B21] Chen Z Gore BB Long H Ma L Tessier-Lavigne M Alternative splicing of the Robo3 axon guidance receptor governs the midline switch from attraction to repulsion Neuron 2008 58 325 332 10.1016/j.neuron.2008.02.016 18466743

[B22] Jaworski A Long H Tessier-Lavigne M Collaborative and specializaed functions of Robo1 and Robo2 in spinal commissural axon guidance J Neurosci 2010 30 9445 9453 10.1523/JNEUROSCI.6290-09.2010 20631173 PMC6632452

[B23] Chen H Chedotal A He Z Goodman CS Tessier-Lavigne M Neuropilin-2, a novel member of the neuropilin family, is a high affinity receptor for the semaphorins Sema E and Sema IV but not Sema III Neuron 1997 19 547 559 10.1016/S0896-6273(00)80371-2 9331348

[B24] Kolodkin AL Levengood DV Rowe EG Tai YT Giger RJ Ginty DD Neuropilin is a semaphorin III receptor Cell 1997 90 753 762 10.1016/S0092-8674(00)80535-8 9288754

[B25] Yoshida Y Semaphorin signaling in vertebrate neural circuit assembly Front Mol Neurosci 2012 5 71 22685427 10.3389/fnmol.2012.00071 PMC3368236

[B26] Zou Y Stoeckli E Chen H Tessier-Lavigne M Squeezing axons out of the gray matter: a role for Slit and Semaphorin proteins from midline and ventral spinal cord Cell 2000 102 363 375 10.1016/S0092-8674(00)00041-6 10975526

[B27] Nawabi H Briancon-Marjollet A Clark C Sanyas I Takamatsu H Okuno T Kumanogoh A Bozon M Takeshima K Yoshida Y Moret F Abouzid K Castellani V A midline switch of receptor processing regulates commissural axon guidance in vertebrates Genes & Devel 2010 24 396 410 10.1101/gad.542510 20159958 PMC2816738

[B28] Parra LM Zou Y Sonic hedgehog induces response of commissural axons to Semaphorin repulsion during midline crossing Nat Neurosci 2010 13 29 35 10.1038/nn.2457 19946319

[B29] Charoy C Nawabi H Reynaud F Derrington E Bozon M Wright K Falk J Helmbacher F Kindbeiter K Castellani V GDNF activates midline repulsion by Semaphorin3B via NCAM during commissural axon guidance Neuron 2012 75 1051 1066 10.1016/j.neuron.2012.08.021 22998873

[B30] Caspary T Anderson KV Patterning cell types in the dorsal spinal cord: what the mouse mutants say Nat Rev Neurosci 2003 4 289 297 10.1038/nrn1073 12671645

[B31] Sakai N Insolera R Sillitoe RV Shi SH Kaprielian Z Axon sorting within the spinal cord marginal zone via Robo-mediated inhibition of N-cadherin controls spinocerebellar tract formation J Neurosci 2012 32 15377 15387 10.1523/JNEUROSCI.2225-12.2012 23115176 PMC3511830

[B32] Kadison SR Murakami F Matise MP Kaprielian Z The role of floor plate contact in the elaboration of contralateral commissural projections within the embryonic mouse spinal cord Dev Biol 2006 296 499 513 10.1016/j.ydbio.2006.06.022 16854408

[B33] Augsburger A Schuchardt A Hoskins S Dodd J Butler S BMPs as mediators of roof plate repulsion of commissural neurons Neuron 1999 24 127 141 10.1016/S0896-6273(00)80827-2 10677032

[B34] Imondi R Wideman C Kaprielian Z Complementary expression of transmembrane ephrins and their receptors in the mouse spinal cord: a possible role in constraining the orientation of longitudinally projecting axons Development 2000 127 1397 1410 10704386 10.1242/dev.127.7.1397

[B35] Kapfhammer JP Xu H Raper JA The detection and quantification of growth cone collapsing activities Nat Protoc 2007 2 2005 2011 10.1038/nprot.2007.295 17703212

[B36] Tran TS Phelps PE Axons crossing in the ventral commissure express L1 and GAD65 in the developing rat spinal cord Dev Neurosci 2000 22 228 236 10.1159/000017445 10894986

[B37] Tran TS Cohen-Cory S Phelps PE Embryonic GABAergic spinal commissural neurons project rostrally to mesencephalic targets J Comp Neurol 2004 475 327 339 10.1002/cne.20166 15221949

[B38] Tamamaki N Yanagawa Y Tomioka R Miyazaki J Obata K Kaneko T Green fluorescent protein expression and colocalization with calretinin, parvalbumin, and somatostatin in the GAD67-GFP knock-in mouse J Comp Neurol 2003 467 60 79 10.1002/cne.10905 14574680

[B39] Tran TS Rubio ME Clem RL Johnson D Case L Tessier-Lavigne M Huganir RL Ginty DD Kolodkin AL Secreted semaphorins control spine distribution and morphogenesis in the postnatal CNS Nature 2009 462 1065 1069 10.1038/nature08628 20010807 PMC2842559

[B40] Beal JA Bice TN Neurogenesis of spinothalamic and spinocerebellar tract neurons in the lumbar spinal cord of the rat Brain Res Dev Brain Res 1994 78 49 56 10.1016/0165-3806(94)90008-6 8004773

[B41] Plump AS Erskine L Sabatier C Brose K Epstein CJ Goodman CS Mason CA Tessier-Lavigne M Slit1 and Slit2 cooperate to prevent premature midline crossing of retinal axons in the mouse visual system Neuron 2002 33 219 232 10.1016/S0896-6273(01)00586-4 11804570

[B42] Rabe N Gezelius H Vallstedt A Memic F Kullander K Netrin-1-dependent spinal interneuron subtypes are required for the formation of left-right alternating locomotor circuitry J Neurosci 2009 29 15642 15649 10.1523/JNEUROSCI.5096-09.2009 20016078 PMC6666172

[B43] Lai HC Klisch TJ Roberts R Zoghbi HY Johnson JE In vivo neuronal subtype-specific targets of Atoh1 (Math1) in dorsal spinal cord J Neurosci 2011 31 10859 10871 10.1523/JNEUROSCI.0445-11.2011 21795538 PMC3153066

[B44] Hernandez-Miranda LR Cariboni A Faux C Ruhrberg C Cho JH Cloutier JF Eickholt BJ Parnavelas JG Andrews WD Robo1 regulates semaphorin signaling to guide the migration of cortical interneurons through the ventral forebrain J Neurosci 2011 31 6174 6187 10.1523/JNEUROSCI.5464-10.2011 21508241 PMC3088089

[B45] Stein E Tessier-Lavigne M Hierarchical organization of guidance receptors: silencing of Netrin attraction by Slit through a Robo/DCC receptor complex Science 2001 291 1928 1938 10.1126/science.1058445 11239147

[B46] Yaron A Huang PH Cheng HJ Tessier-Lavigne M Differential requirement for Plexin-A3 and -A4 in mediating responses of sensory and sympathetic neurons to distinct class 3 Semaphorins Neuron 2005 45 513 523 10.1016/j.neuron.2005.01.013 15721238

[B47] Tran TS Kolodkin AL Bharadwaj R Semaphorin regulation of cellular morphology Annu Rev Cell Dev Biol 2007 23 263 292 10.1146/annurev.cellbio.22.010605.093554 17539753

[B48] Bonanomi D Pfaff SL Motor axon pathfinding Cold Spring Harb Perspect Biol 2010 2 a001735 10.1101/cshperspect.a001735 20300210 PMC2829954

[B49] Kao TJ Law C Kania A Eph and ephrin signaling: lessions learned from spinal motor neurons Semin Cell Dev Biol 2012 23 83 91 10.1016/j.semcdb.2011.10.016 22040916

[B50] Giger RJ Cloutier JF Sahay A Prinjha RK Levengood DV Moore SE Pickering S Simmons D Rastan S Walsh FS Kolodkin AL Ginty DD Geppert M Neuropilin-2 is required in vivo for selective axon guidance responses to secreted semaphorins Neuron 2000 25 29 41 10.1016/S0896-6273(00)80869-7 10707970

[B51] Jevince AR Kadison SR Pittman AJ Chien CB Kaprielian Z Distribution of EphB receptors and ephrin-B1 in the developing vertebrate spinal cord J Comp Neurol 2006 497 734 750 10.1002/cne.21001 16786562 PMC2637817

[B52] Bravo-Ambrosio A Mastick G Kaprielian Z Motor axon exit from the mammalian spinal cord is controlled by the homeodomain protein Nkx2.9 via Robo-Slit signaling Development 2012 139 1435 1446 10.1242/dev.072256 22399681 PMC3308178

